# The effects of a single aerobic exercise session on mood and neural emotional reactivity in depressed and healthy young adults: A late positive potential study

**DOI:** 10.1111/psyp.14137

**Published:** 2022-07-05

**Authors:** Tomasz S. Ligeza, Marcin Maciejczyk, Miroslaw Wyczesany, Markus Junghofer

**Affiliations:** ^1^ Psychophysiology Laboratory, Institute of Psychology Jagiellonian University Kraków Poland; ^2^ Department of Physiology and Biochemistry, Faculty of Physical Education and Sport University of Physical Education Kraków Poland; ^3^ Institute for Biomagnetism and Biosignal analysis University of Muenster Muenster Germany; ^4^ Otto Creutzfeldt Center for Cognitive and Behavioral Neuroscience University of Muenster Muenster Germany

**Keywords:** aerobic exercise, depression, EEG, emotional pictures, emotional reactivity, LPP

## Abstract

Depression has been characterized by lowered mood and unfavorable changes in neural emotional reactivity (altered brain responses to emotional stimuli). Physical exercise is a well‐established strategy to improve the mood of healthy and depressed individuals. Increasing evidence suggests that exercise might also improve emotional reactivity in healthy adults by increasing or decreasing brain responses to positive or negative stimuli, respectively. It is unknown, however, if exercise could also benefit emotional reactivity in depressed individuals. We investigated the effects of a single aerobic exercise session on mood and emotional reactivity in 24 depressed and 24 matched healthy young adults. Self‐reported mood and neural reactivity to emotional pictures (indexed by the EEG late positive potential, LPP) were assessed before and after two experimental protocols: exercise (36 min of moderate‐intensity exercise at 75% of maximal heart rate) and seated rest condition (36 min). In the healthy control group, exercise improved self‐reported mood and neural emotional reactivity (increasing LPP to positive pictures). In the depressed group, exercise improved self‐reported mood; however, it did not affect neural emotional reactivity. Additional analyses performed on both groups revealed that exercise‐induced changes in emotional reactivity are associated with the severity of depressive symptoms: the effectiveness of exercise in improving emotional reactivity decreases with the severity of depressive symptoms. Overall, the study further strengthens the claim of a beneficial role of exercise on mood and emotional reactivity. It also suggests that a single aerobic exercise session might have a limited influence on neural emotional reactivity in depressed individuals.

## Abstract 2

Acute exercise positively influences neuronal emotional reactivity in healthy adults by increasing or decreasing brain responses to positive or negative stimuli, respectively; however, it is still unclear whether this applies to individuals who are depressed. Our study provides new insights into the effectiveness of exercise in influencing neural emotional responsiveness in depressed individuals.

## 1 INTRODUCTION

Physical exercise is becoming an increasingly popular strategy to improve emotional functioning. Even one session of exercise (hereafter exercise) benefits mood. Recent evidence has shown that exercise may also improve neural emotional reactivity by increasing the brain's response to positive stimuli or inhibiting responses to negative stimuli in healthy adults. As emotional reactivity is altered in depressed individuals, exercise might be a promising intervention to counteract these adverse changes. The study's main aim is to shed more light on the effects of exercise on neuronal emotional responsiveness in depressed individuals.

### 1.1 Depression and emotional reactivity

Depression is one of the most prevalent, debilitating, and highly recurrent forms of psychopathology and is manifested by feelings of sadness or loss of interest in once‐enjoyed activities (Kessler et al., 2005). The key indications of depression are lowered mood and anhedonia (the inability to experience pleasure and reduced reactivity to rewards). As is consistent with disturbed mood, depression is also related to negative patterns of responses to emotional stimuli (emotional reactivity). Although both mood and emotions are types of affective experience, mood is less specific, less intense, and longer‐lasting than emotions (Ekkekakis, 2012).

Three different models describe disturbances in emotional reactivity in depressed individuals. The *low positive emotion model* assumes that depressed individuals show substantial and selective deficits in responding to pleasant stimuli, thus their positive mood is dampened (Clark et al., 1994; Raes et al., 2012). *The heightened negative emotion model* assumes that depressed individuals are more responsive to unpleasant stimuli, thus causing a depressed mood (e.g., Clark & Watson, 1991; Nitschke et al., 2001). Together, both models conform to cognitive theories of depression (e.g., Beck's theory), which assume biased perception marked by the selective filtering out of positive information and the exaggeration of negative information (Beck, 1976).

To explain changes in emotional reactivity in depressed individuals, theorists also formulated another perspective called the *emotion context insensitivity model (ECI)* (Rottenberg et al., 2005). *ECI* is rooted in an evolutionary perspective of depression and states that depressed individuals display reduced emotional reactivity to both pleasant and unpleasant stimuli. In this context, depressed mood represents a defensive mechanism that promotes disengagement from a potentially dangerous environment, yet it causes depressed individuals to perceive their world as flat, dull, and empty (Jackson, 1986). According to this model, similarly to the *low positive emotion model*, depressed individuals show decreased emotional reactivity to positive stimuli; however, contrary to the *heightened negative emotion model*, they also show decreased emotional reactivity to negative stimuli.

### 1.2 LPP as a reliable measure of neural emotional reactivity

The patterns of emotional reactivity proposed by the three models described above have been empirically tested across multiple levels of analysis, including self‐report, behavioral, and psychophysiological measures (e.g., Benning & Ait Oumeziane, 2017). Neural measures objectively measure the emotional response of the brain. The most recognized measure of neural emotional reactivity is the late positive potential (LPP), which is derived from electroencephalography (EEG) (Olofsson et al., 2008). Compared with other measures (especially when contrasted with self‐reports), the high temporal resolution of EEG allows the dynamic nature of emotional responsiveness to be captured. LPP is a late‐latency component that develops 400–500 ms after stimulus onset; it lasts up to several seconds and demonstrates a central posterior topography (which typically accumulates around the Pz electrode). LPP indexes sustained attention toward motivationally (emotionally) salient stimuli such that it is larger in response to more emotionally laden or salient stimuli (Schupp et al., 2000, 2006).

Experimental support in favor of all of these three models of emotional reactivity can be found in the literature. In line with the *low positive emotion model*, reduced LPP to positive vs. neutral pictures has been associated with depressive symptoms (MacNamara et al., 2016; Sandre et al., 2019; Weinberg et al., 2016). Lending support to the *heightened negative emotion model*, evidence showed increased LPP to negatively valenced pictures in depressed people (Auerbach et al., 2015; Burkhouse et al., 2017; Speed et al., 2015). Finally, increasing evidence suggests that depressive symptoms might be linked to generally reduced LPP in response to pleasant and unpleasant stimuli, thus supporting the *ECI model* (e.g., Foti et al., 2010; Klawohn et al., 2021). The factors determining support for a specific model are not fully understood. They might relate to differences in experimental procedures (e.g., presentation of pictures vs. words as stimuli), individual differences of participants across studies (e.g., age and personality traits), as well as coexisting mental disorders in participants, for example, high levels of anxiety (Kujawa et al., 2015; MacNamara et al., 2016). Therefore, changes in neuronal emotional reactivity in depression can take any of the forms described by the three models, and LPP might index the possible changes predicted by all three models.

### 1.3 Exercise as a possible intervention to counteract unfavorable changes in mood and emotional reactivity

Interventions aimed at counteracting unfavorable changes in mood (making depressed individuals feel better) and emotional reactivity (making depressed individuals process negative stimuli less intensely/positive stimuli more intensely, or increasing their overall emotional reactivity) may serve to improve the health of depressed individuals. Current depression treatments focus primarily on the use of psychopharmacology. Antidepressant treatment can ameliorate some adverse changes in emotional reactivity, specifically in severely disordered patients, thus promoting reactivity to positive stimuli (Harmer & Cowen, 2013). However, long‐term medication might lead to potentially harmful side effects.

Physical exercise might be an alternative low‐cost, no side‐effects preventive strategy, or treatment for mood disorders. Many studies have shown that exercise improves mood states in healthy adults. In fact, even one aerobic exercise session (such as 30 min of jogging or biking) alleviates negative (Asmundson et al., 2013) and enhances positive mood (Reed & Ones, 2006). Acute aerobic exercise also has a positive effect on the mood of depressed individuals. In their seminal work, Bartholomew et al. (2005) found that exercise improved positive (positive well‐being, vigor) and attenuated negative (psychological distress, depression, confusion, fatigue, tension, anger) mood states of depressed participants. However, in this study exercise showed additional benefits over the control condition (quiet rest) for positive mood states only. Similar exercise‐induced improvements that were limited to the positive mood states of depressed patients can be found in the studies of Frühauf et al. (2016) (excitement, activation) and Legrand et al. (2018) (energy). Beneficial effects of exercise for both the positive and negative mood states of depressed individuals that were greater than the effects of the control conditions (i.e., reading) were reported by Brupbacher et al. (2021) as well as Niedermeier et al. (2021). Studies that focused predominantly on the effects of exercise on negative mood states in depression were rather inconclusive: whereas one study showed that exercise decreased depressive mood states (Meyer et al., 2016), two other studies from the same research group failed to show this effect to be greater than the effects of the control condition (i.e., quiet rest) (Meyer et al., 2019; Perez et al., 2020).

Together, the available evidence consistently indicates that exercise can increase positive and reduce negative self‐reported mood states in healthy and depressed individuals, although the effects of exercise are not always greater than the effects of a control condition. However, much less is known about how exercise could influence emotional reactivity. Given the overall beneficial effects of exercise on mood, it might be expected that exercise would also benefit emotional reactivity in a mood‐congruent manner. Research shows that a positive mood might boost emotional responsiveness to positive stimuli (e.g., Voelkle et al., 2014). Similarly, negative mood may lead to greater reactivity to negative stimuli (such a negativity bias is often observed in depressed individuals). Therefore, it can be expected that exercise should have overall positive effects on affective functioning, including improvements in mood and emotional responsiveness.

Increasing evidence suggests that exercise might indeed benefit emotional reactivity. To date, several studies have investigated the relationship between exercise and the brain's reactivity to emotional stimuli in healthy adults (Brush et al., 2020, 2021; Ligeza et al., 2020; Schmitt et al., 2019; Tartar et al., 2018; Thom et al., 2019). These studies employed a similar experimental scheme and focused on the influence of one exercise session (acute exercise) on reactivity to emotional pictures. Participants completed a session of exercise and a session of “seated rest” (control condition). During each session, participants were presented with emotional pictures and asked to watch them passively. To measure neural emotional reactivity, most of these studies used LPP.

These studies predicted relatively reduced LPP in response to negative pictures and/or relatively increased LPP in response to positive pictures after exercise (compared with the control condition). Although Thom et al. (2019) found that exercise did not change LPP in response to anger‐evoking pictures, Tartar et al. (2018) reported that exercise reduced the LPP for negative pictures; this suggests decreased emotional reactivity to negative pictures after exercise. In turn, the two studies of Brush et al. (2020, 2021) indicated that LPP in response to positive pictures was potentiated following exercise, suggesting increased reactivity to positive pictures after exercise The studies of Brush et al. (2020, 2021) either did not test the influence of exercise on reactivity to negative pictures (Brush et al., 2021) or found no effect of exercise on reactivity to negative pictures (Brush et al., 2020).

Studies testing emotional reactivity after exercise using measures of brain responses that are complementary to EEG provided additional support for the LPP data. By utilizing source reconstructions of brain activity measured by magnetoencephalography (MEG), the recent study of Ligeza et al. (2020) tested the influence of exercise on the processing of positive and negative pictures. The results showed that brain reactivity to positive pictures increased after exercise (compared to the rest condition), whereas brain reactivity to negative pictures was slightly attenuated. Finally, an fMRI study that tested the influence of exercise on the processing of emotional faces (Schmitt et al., 2019) revealed that exercise decreased the brain activation induced by fearful faces in face‐processing brain regions. Together, studies on the effects of exercise on emotional reactivity in healthy adults suggest that exercise has substantial and positive effects on emotional reactivity.

### 1.4 Current evidence on the role of exercise in counteracting unfavorable changes in emotional reactivity in depressed individuals

The positive effects of exercise on emotional reactivity in healthy adults seem to be particularly interesting in the context of interventions aimed at counteracting adverse changes in emotional reactivity in depressed individuals. So far, however, only two studies have tested this possibility.

Both studies focused on the influence of exercise on LPP in response to emotional pictures. Brush et al. (2020) tested the effects of exercise (vs. seated rest) on mood, and reactivity to positive, neutral, and negative pictures in individuals reporting variable symptoms of depression. In this study, exercise (compared to a seated rest condition) improved mood, and increased LPP in response to positive pictures, regardless of the severity of depressive symptoms. Additionally, this study showed correlations between exercise‐induced changes in mood and LPP in response to pictures. Mood improvements were associated with greater LPP in response to positive pictures and lower LPP in response to negative pictures. This confirms an overall positive effect of exercise on emotional functioning: both on subjective mood and emotional responsiveness levels. A second study by Brush et al. (2021) tested the effects of exercise on emotional reactivity in a group of 43 adults with a current or lifetime history of depression and in a group of 18 never‐depressed healthy adults. Similar to the first study, the results showed increased positive reactivity after exercise (compared to before exercise). However, the beneficial effects of exercise were observed in the group of healthy adults only, while exercise did not change LPP in response to positive pictures among participants with current or lifetime depression. Additional analysis on subgroups of depressed participants suggested that a lack of exercise‐induced effects depends on the depression type. The lack of improvement in response to exercise concerned a subgroup of depressed participants who exhibited impaired mood reactivity, which is understood as a lack of reactivity to usually pleasurable stimuli. In contrast, those with intact mood reactivity showed an increase in LPP to positive pictures.

To summarize, while the first study provided evidence that exercise increases responses to positive stimuli regardless of depressive symptoms, the second study suggested that exercise might positively influence only the emotional reactivity of depressed individuals with intact mood reactivity. These two studies provide essential insights into the potential role of exercise in affecting emotional reactivity in depressed individuals. However, as both studies showed different results, more research is needed to understand the role of exercise in affecting the emotional responsiveness of depressed individuals.

Most importantly, as suggested by the study of Brush et al. (2021), considering additional individual characteristic variables that could mediate the relationship between exercise and changes in emotional reactiveness might be essential to understand the role of exercise in improving emotional responsiveness. Moreover, to increase the robustness of results, some refinements to the previous procedures are feasible. Brush et al. (2020) utilized post‐test measures, testing emotional reactivity only after exercise and control (rest) conditions. Including additional pre‐test measurements could reduce the day‐to‐day variability of measurements, thus increasing data reliability. Although Brush et al. (2021) used both pre‐and post‐test measures, a control (rest) condition was not utilized. A control condition seems essential to distinguish the effects of exercise from other factors that could vary between pre‐ and post‐test measures (e.g., repeated exposure to emotional pictures). Finally, similarly to Brush et al. (2020) but differently from Brush et al. (2021), assessing emotional responsiveness to both positive and negative pictures could shed more light on the nature of the influence of exercise on emotional responsiveness.

### 1.5 Current study and hypotheses

In the current study, we aimed to investigate the impact of a single exercise session on mood and emotional reactivity in depressed and healthy young adults. Our primary goal was to test the influence of exercise on neural responsiveness in a depressive group. Nevertheless, our study also aimed to provide further evidence to support the claim that a session of exercise improves mood in both groups, and it improves neural emotional responsiveness in the healthy group.

Taking into account the potentially limiting factors of the previous studies outlined above, in this study, we assess neural emotional reactivity in response to negative and positive pictures (c.a. Brush et al., 2021) before and after exercise (c.a. Brush et al., 2020) as well as before and after a control condition (pre‐ and post‐test measures) (c.a. Brush et al., 2020). Since the study of Brush et al. (2021) indicated the potential role of individual variables in explaining exercise‐induced changes in emotional reactivity, we additionally measured several individual characteristics in both groups, including severity of depressive symptoms, anxiety levels, emotional control tendencies, IQ level, and perceived stress levels. We additionally measured variables related to physical exercise: participants' daily caloric expenditure and an indirect estimate of their physical fitness. Finally, we also measured changes in mood in response to exercise, as mood reactivity could potentially predict the effectiveness of exercise in affecting emotional reactivity (Brush et al., 2021).

In line with previous evidence, we expected that exercise would positively affect mood (increase the positive and decrease the negative mood domains). Based on previous studies on the influence of exercise on emotional reactivity in healthy adults, we expected that emotional reactivity to pictures (as measured by LPP) would change after exercise (compared with before exercise) (e.g., Brush et al., 2020; Ligeza et al., 2020; Tartar et al., 2018). Specifically, we predict that LPP in response to positive pictures should increase after exercise, whereas LPP in response to negative pictures should decrease. In the light of the unclear evidence on the impact of exercise on emotional reactivity in depression, we assumed the same positive impact of exercise in depressed individuals as in healthy adults. Regarding the dependencies between exercise‐induced changes in emotional reactiveness and individual characteristics, we took an exploratory approach and did not formulate specific hypotheses. However, based on Brush et al.’s (2021) previous study, an exercise‐induced mood increase would be expected to be associated with benefits in emotional responsiveness.

## 2 METHOD

### 2.1 Participants

Participants were recruited via advertisements posted on local online advertising services and social media portals of Jagiellonian University in Krakow, Poland. The sample was drawn from university students in Krakow, Poland, local communities of psychological support centers, and other city residents who qualified for the study. Inclusion criteria were as follows: 20–40 years old, normal or corrected‐to‐normal vision, no history of neurological disorders, and good health. Participants meeting these initial preconditions and interested in volunteering in the project were contacted via email and asked to complete an online pre‐screening questionnaire. For the pre‐screening, we used an online survey that consisted of questions about basic demographic data (sex, age, education, height, weight); Beck's Depression Inventory (BDI‐II; to measure the severity of depressive symptoms initially) (Beck et al., 1996); The Physical Activity Readiness Questionnaire (PAR‐Q; to verify if participants can safely engage in physical activity) (Thomas et al., 1992); and the Seven‐Days Physical Activity Recall Questionnaire (SDPARQ; to assess the physical activity levels of participants) (Washburn et al., 2003).

Based on the survey output, participants were selected for the further recruitment process according to the following criteria: (1) at least mild depressive disorders or no depressive disorder (reported score in BDI‐II ≥15 or < 10 points, respectively^1^) and (2) no contraindication to engage in physical activity (answer *no* to all questions in PAR‐Q). Participants meeting these criteria were invited for a qualifying meeting.

During the qualifying meeting, an experienced clinical psychologist assessed participants for the presence and severity of depressive disorders. The presence of depressive episodes, persistent depressive disorder, or recurrent depressive disorder was diagnosed using the Mini‐International Neuropsychiatric Interview (Sheehan et al., 1998) according to the *Diagnostic and Statistical Manual of Mental Disorders* (5th ed.; DSM‐5; American Psychiatric Association, 2013) (version 7.0.2 of MINI), whereas the severity of depressive symptoms was assessed using a structured interview (Hamilton Rating Scale for Depression, HRSD, 21‐item version; also abbreviated in the literature to HDRS [Hamilton Depression Rating Scale] or Ham‐D) (Hamilton, 1960). During the diagnostic session, the severity of depressive disorders was determined using a different tool (HRSD) than in the pre‐screening (BDI‐II) to avoid a potential bias in participants' responses related to re‐answering the same questions. An additional interview was conducted to perform a functional diagnosis that was intended to clarify possible doubts that may have arisen during the meeting, such as questions about the context of depressive disorders, substance use, comorbidities, or any doubts regarding readiness for physical exercise.

Participants were assigned to the depression (DEP) group or healthy control (HC) group. The inclusion criterion for the DEP group was the confirmed presence of depressive disorders with at least mild severity (at least 12 points in HRSD); for the HC group, the inclusion criterion was confirmation of no depressive disorders and less than 7 points in HRSD. After qualifying for the study, each participant was given a brief introduction and was asked to provide informed written consent. The experimental procedure complied with the directives of the Helsinki Declaration and was approved by the ethical board of Jagiellonian University in Krakow (Poland).

**FIGURE 1:**
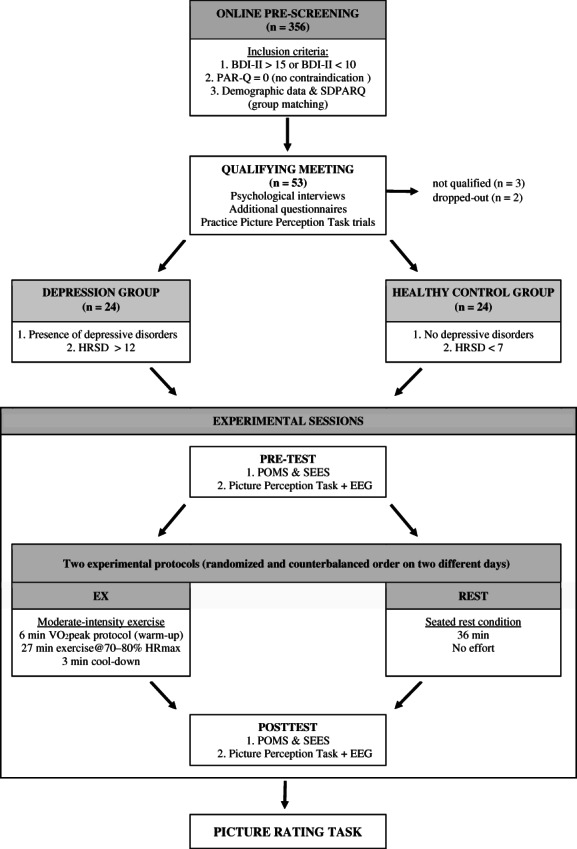
The study diagram. *BDI‐II*, Beck depression inventory; *PAR‐Q*, physical activity readiness questionnaire; *SDPARQ*, seven‐days physical activity recall questionnaire; *HRDS*, Hamilton rating scale for depression; *POMS*, profile of mood state scale; *SEES*, subjective exercise experience scale.

After several participants had completed the experimental procedure, subsequent participants were recruited based on additional criteria to match participants in the DEP group and participants in the HC group in terms of gender, age, and (as closely as possible) reported physical activity levels (SDPARQ). Based on our previous study on the influence of exercise on emotional reactivity (Ligeza et al., 2020), we aimed to test 24 participants in each group (48 in total). Out of the 356 respondees who completed the online survey, 53 were invited to the qualifying meeting. Three individuals did not qualify for the study because they failed to meet the assumptions regarding depressive disorders. In the case of excluded participants, the BDI‐II scores indicated depressive symptoms during pre‐screening, but a psychologist did not confirm the presence of depression during the standardized interview. Two participants withdrew after qualification for the study. Finally, 48 participants underwent the whole testing procedure (24 in the DEP group and 24 in the HC group). Participants' characteristics are presented in Table 1, whereas the overview of the recruitment process (and experimental design) is provided in Figure 1.

**TABLE 1:** Participants characteristics (M ± SD)

	Depression group (DEP)	Healthy control group (HC)	*p* *	Total
*n* (females)	24 (12)	24 (12)	1	48 (24)
Age	24.50 ± 5.18	24.50 ± 5.18	1	24.50 ± 5.12
Race/ethnicity Caucasian/Polish	24 (100%)	24 (100%)	1	48 (100%)
Education				
Below high school	2 (8.3%)	1 (4.2%)	n/a	3 (6.25%)
High school	5 (20.8%)	5 (20.8%)	n/a	10 (20.8%)
Undergraduate	11 (45.8%)	12 (50%)	n/a	23 (47.9%)
Graduate	6 (25%)	6 (25%)	n/a	12 (25%)
BMI (kg/m^2^)	23.54 ± 4.51	22.80 ± 3.20	.519	23.17 ± 3.89
SDPARQ (kcal/day)	2469 ± 272	2430 ± 625	.780	2449 ± 477
VO_2_peak (ml·kg^−1^·min^−1^)	39.67 ± 10.27	41.41 ± 9.10	.538	40.54 ± 9.64
Depression (BDI‐II)	22.54 ± 5.78	3.50 ± 2.72	**<.001**	13.02 ± 10.60
Depression (HRSD)	17.58 ± 3.31	2.50 ± 1.96	**<.001**	10.04 ± 8.08
Current comorbidity (%)	9 (38%)	n/a	n/a	n/a
Psychotropic medication use (%)	6 (25%)	n/a	n/a	n/a
Anxiety (STAI trait)	49.376 ± 7.80	34.63 ± 7.91	**<.001**	42.00 ± 10.77
Perceived stress (PSS)	17.92 ± 7.16	17.96 ± 7.50	.984	17.94 ± 7.25
IQ (sRAMP)	11.75 ± 3.78	11.92 ± 4.06	.884	11.83 ± 3.88
Cognitive reappraisal (ERQ)	4.91 ± 1.27	4.44 ± 1.23	.197	4.67 ± 1.26
Expressive suppression (ERQ)	3.99 ± 1.42	3.72 ± 1.37	.505	3.85 ± 1.39

Abbreviations: BDI‐II, Beck Depression Inventory; BMI, body mass index; ERQ, Emotion Regulation Questionnaire; HRSD, Hamilton Rating Scale for Depression; PSS, Perceived Stress Scale; SDPARQ, Seven‐Days Physical Activity Recall Questionnaire; sRAMP, shortened version of Raven's Advanced Progressive Matrices test; STAI, Spielberger State–Trait Anxiety Inventory; VO_2_peak, peak oxygen uptake.

* Significance of the difference between depression (DEP) and healthy control (HC) group (unpaired Student's *t‐test*).

### 2.2 Questionnaires and tests

In addition to the questionnaires used during the online pre‐screening and qualifying meeting (BDI‐II, I‐PAQ, SDPARQ, HRSD; see Participants section for more details), participants who qualified for the study completed an additional set of questionnaires and an IQ test. Additional questionnaires included the Spielberger State–Trait Anxiety Inventory (STAI, Spielberger et al., 1983) (a measure of anxiety level understood as a personal trait); the Perceived Stress Scale (PSS, Cohen et al., 1983) (a measure of subjective perception of stress); and the Emotion Regulation Questionnaire (ERQ, Gross & John, 2003) (a measure of individual differences in the habitual use of two emotion regulation strategies: expressive suppression and cognitive reappraisal). The choice of the ERQ questionnaire was justified by the fact that habitual use of reappraisal is associated with the decreased amplitude of the LPP component in response to emotional pictures (e.g., Harrison & Chassy, 2019). Potential differences in this variable across the two groups could be confounding factors in the analyses of LPP data. Finally, the intelligence quotient (IQ) was measured using a shortened version of Raven's Advanced Progressive Matrices test (sRAPM, see: Marzecová et al., 2013 for a detailed description). These data allowed us to collect more information on participants' background characteristics, compare the two tested groups, and explore the relationships between participants' characteristics and exercise‐induced changes in emotional reactivity.

### 2.3 Experimental measures

#### 2.3.1 Picture perception task

The picture perception task consisted of 480 trials, which included six repetitions of 80 pictorial stimuli presented in six runs with one‐minute breaks in between. Participants were asked to view the emotional scenes passively without additional tasks. The 80 stimuli depicted 40 pleasant scenes (erotic scenes, sports, happy people) and 40 unpleasant scenes (threatening scenes, mutilation, sad people).^2^ The erotica portrayed attractive, heterosexual couples engaging in normative sexual activity. The sport scenes depicted people engaging in different sports, including skiing, swimming, and track. Happy people included romantic couples, families, and children showing joy in everyday situations. Threatening scenes included people engaged in menacing or violent actions toward other people or toward the camera lens. Scenes in the mutilation category depicted graphic images of knife wounds, mangled limbs, and exposed wounds. Sad people included couples, families, and children showing sadness or suffering.^3^ To optimize our investigation of the effect of interest (responses to negative and positive pictures), we deliberately did not include neutral pictures in the procedure. This limited the additional variance of low‐arousal neutral pictures.

Images were balanced across the categories so as to be statistically equivalent in luminosity and physical complexity, indexed by 90% quality JPEG files using GIMP 2.8 (www.gimp.org). The grayscale scenes were presented at 800 × 600 resolution using PsychoPy v2021.2.1 software (Peirce et al., 2019) on a computer with a 27″ high‐definition screen. Stimuli were presented for 1000 ms with a randomly chosen inter‐stimulus interval of between 1000 and 2000 ms. A red fixation dot presented at the center of the screen was continuously shown to reduce eye movements. Stimulus order within each block was randomized, but the transition probabilities between emotional categories were equal in all blocks, with equal transition probabilities between emotional categories.

After completing all the experimental procedures, participants rated the hedonic valence and emotional arousal of all pictures presented during the procedure using a computerized version of the Self‐Assessment Manikin (Bradley & Lang, 1994). The responses were given using two bipolar visual analog scales (VAS), which ranged from positive to negative (for the hedonic valence of the pictures), and from “not arousing” to “very arousing” (for the emotional arousal of the pictures). Then, responses were decoded to numerical values ranging from 0 (for the most negative/least arousing pictures) to 100 (for the most positive/most arousing pictures). The negative and positive sets of pictures differed in their ratings of hedonic valence [*M* = 21.19, *SD =* 10.30 for the negative set; *M* = 74.29, *SD =* 11.94 for the positive set; *t*(47) = 17.59, *p* < .01]; the two sets did not differ with regard to emotional arousal [*M* = 57.31, *SD =* 13.47 for the negative set *M* = 59.49, *SD =* 13.18 for the positive set; *t*(47) = 1.53, *p* = .15].

#### 2.3.2 Mood assessment

During the experimental procedures, participants' mood was assessed using a modified version of the Short Form of the Profile of Mood State Scale (POMS‐SF), developed by Grove and Prapavessis (1992), and the Subjective Exercise Experience Scale (SEES) (McAuley & Courneya, 1994).

The used version of POMS consists of 40 items (adjectives referring to feelings) that are rated on a 5‐point Likert scale ranging from “Not at all” to “Extremely.” Individual items form seven subscales. Two of the scales measure positive mood: (1) Esteem‐related Affect (ERA, *Cronbach's* α^4^ = 0.76); (2) Vigor (VIG, *Cronbach's* α = 0.92). Five of the scales measure negative mood: (1) Tension (TEN, *Cronbach's* α = 0.92); (2) Anger (ANG, *Cronbach's* α = 0.93); (3) Fatigue (FAT, *Cronbach's* α = 0.89); (4) Depression (DEP, *Cronbach's* α = 0.95); (5) Confusion (CON, *Cronbach's* α = 0.79). The Global Total Mood Disturbance (TMD) scale is calculated by summing the totals of the negative subscales (TEN + ANG + FAT + DEP + CON) and then subtracting the totals of the positive subscales (ERA, VIG).

SEES measures global psychological responses to exercise. It is a 12‐item questionnaire scored on a 7‐point Likert‐like scale, which is anchored with “Not at all” and “Very much so.” The SEES provides three subscales: (1) Psychological Well‐Being (PWB, *Cronbach's* α = 0.89 in this study); (2) Psychological Distress (PD, *Cronbach's* α = 0.91); and (3) Fatigue (F, *Cronbach's* α = 0.94).

### 2.4 Experimental protocols

The present study consisted of two experimental protocols in two separate experimental sessions: Exercise (EX) and Seated Rest Condition (REST). Additionally, each participant underwent the VO_2_peak protocol to assess their physical fitness during the EX protocol. The EX and VO_2_peak protocols were administered using a cycle Ergometer (Ergoline, Germany). During both experimental protocols (EX, REST), participants' heart rate (HR) was measured using an HR belt chest strap (Polar H10, Finland). The study overview is presented in Figure 1.

#### 2.4.1 VO_2_peak (peak oxygen uptake) protocol

Peak oxygen uptake (VO_2_peak) was indirectly estimated using the Ǻstrand‐Rhyming cycle test to assess the physical fitness of participants. This test assumes a linear relationship between HR and VO_2_peak (Astrand & Ryhming, 1954). All participants performed submaximal effort at constant power for 6 min. The power output was adjusted to the physical capability of each participant so that they should reach the target HR zone of 115–135 bpm at the end of the six‐minute test. The mean HR for the last two minutes of the protocol was calculated, and the absolute VO_2_peak value was determined. The pedaling rate during the test was set to 60 rpm. The average load used in the protocol was 65.10 ± 22.09 Watts. The average load did not differ across the Dep and HC groups (62.5 ± 20.41 Watts for the DEP group; 67.70 ± 23.36 Watts for the HC group, *t*[46] = −0.81; *p* = .43). The average HR during the protocol was 111.79 bpm ± 12.93. The average HR during the protocol did not differ across the DEP and HC groups (112 ± 14.5 bpm for the DEP group; 111.5. ± 11.2 bpm for the HC group, *t*[46] = 0.13; *p* = .9). Given the physiological parameters of this test, it should be considered a standard warm‐up before the main effort.

#### 2.4.2 Exercise (EX) and seated REST control (REST) protocols

Intensity and duration for the exercise protocol (EX) were chosen based on previous studies that have shown that moderate‐intensity aerobic exercise lasting 20–40 min improves mood most effectively (Brown et al., 1995, p. 195; Daley & Welch, 2004; Ensari et al., 2017; Meyer et al., 2016). The specific intensity of the exercise was also based on international guidelines, which recommend moderate to vigorous aerobic exercise at 65–95% HRmax for most adults (American College of Sports Medicine, 2018). Therefore, we aimed to use exercise with an overall intensity close to the upper range of moderate exercise (76% HRmax, according to the American College of Sports Medicine, 2018, and The Physical Activity Guidelines for Americans, Office of Disease Prevention and Health Promotion, 2008). Additionally, we used an interval form of exercise to maximize the physiological response to exercise without allowing participants to develop too much fatigue (which could negatively affect mood). Specifically, the higher‐intensity bout of exercise (80% HRmax) was assumed to maximize the appropriate cardiopulmonary response. In contrast, lower‐intensity bouts of exercise (at 70% HRmax) were intended to provide partial recovery. Moreover, the interval form of the exercise was intended to increase the enjoyment of the exercise for the participants, as evidenced by a systematic review and meta‐analysis of studies comparing the effects of moderate‐intensity continuous and high‐intensity interval exercises on mood (Niven et al., 2020). This work indicates that the high‐intensity interval exercise is more enjoyable for the participants due to its alternating nature, making the exercise more engaging. At the same time, moderate‐intensity exercise is more pleasant for the participants because it is of lower intensity, making the exercise less exhausting. By choosing moderate‐intensity interval protocol, we aimed to maximize both enjoyment and pleasantness of the exercise. It should be noted here that the selection of specific exercise parameters (duration, intensity, form) was based on the studies concerning the effects of exercise on mood, as understanding how these parameters could impact neural emotional responsiveness (as indexed by LPP) is still very limited.

Overall, the utilized exercise protocol (EX) consisted of a 6‐min warm‐up (indirect VO_2_peak protocol), 27 min of moderate‐intensity interval exercise, and a 3‐min cool‐down phase (36 min in total). The workload in the warm‐up phase was set according to the VO_2_peak protocol, whereas the workload for the exercise phase was adjusted individually for each participant according to their maximal heart rate (HRmax). HRmax was calculated using the formula HRmax = 208 – (0.7 × age in years) (Tanaka et al., 2001). Six minutes of higher‐intensity exercise (approx. 80% of HRmax) was alternated with 3 min of lower‐intensity exercise (approx. 70% HRmax). During the cooldown, the workload was set to 50% of the average workload used during the exercise phase. Participants were asked to maintain a pedaling rate of 60–70 revolutions per minute during the whole EX protocol. An experimenter (an experienced exercise physiologist) adjusted the bike's resistance on an ongoing basis to meet the participant's predetermined HR zones.

During the control condition, namely the seated rest condition (REST), participants were asked to sit in the exercise room and were instructed to relax or read the provided sport‐related magazines for 36 min.

### 2.5 Experimental procedure

After qualification for the study, each participant was asked to complete additional questionnaires and an IQ test. Afterward, they were familiarized with the experimental task by performing practice trials of the picture perception task (80 trials, one repetition of all pictures presented during experimental sessions). This training session was designed to minimize novelty effects during the experimental sessions and exclude participants from the experiment for whom viewing arousing pictures could cause psychological discomfort (participants were asked to close their eyes and interrupt the session if they felt discomfort). Next, the dates of the two experimental sessions were set. Participants were instructed to avoid any physical activity for 24 h before each laboratory visit, abstain from alcohol consumption 12 h before each visit, and abstain from food for 2 h before each visit. The two experimental sessions were conducted at the same time of day on separate days, 2–7 days apart. The order of protocols during the experimental sessions (EX/REST) was randomized and fully counterbalanced across the final 48 participants.

At the beginning of each experimental session, participants were seated in a softly lit, sound‐attenuated, air‐conditioned EEG chamber; EEG equipment was attached, and they were asked to complete the POMS and SEES questionnaires. Next, participants were presented with the picture perception task, during which EEG was recorded (pre‐test). Then, participants performed one of the experimental protocols in an adjacent exercise room. During the experimental protocol, the participants were already wearing the EEG cap so that the acquisition could start without delay after the protocol. In the last 30 seconds of the EX or REST protocols, participants were asked to rate their perceived exertion during the protocol using the Borg scale (from 6 to 20 points; 6 = no exertion at all; 20 = extremely heavy work) (Borg, 1982) and provide the score immediately after the protocol ended. Approximately 7 min after the protocol ended but not before the participants' HR had returned to baseline HR (±15%), participants completed the POMS and SEES questionnaires and were presented with the picture perception task for a second time (post‐test). After completing both experimental sessions, each participant rated all pictures used in the study and was rewarded for their participation. The overview of the study is provided in Figure 1.

### 2.6 Electrophysiological recordings and preprocessing

The EEG signal was recorded at 256 Hz from 64 Ag/AgCl scalp electrodes, positioned at the standard 10–20 locations, mounted in an elastic cap, using the Biosemi Active Two recording system. The hardware low‐pass filtering was set to 410 Hz with a 5th‐order sinc response filter. To minimize low‐frequency (sweat‐related) artifacts, we used a gel with high saline content. Electrodes were initially referenced online to the Common Mode Sense electrode located at the C1 electrode. The electrode offset was kept within the range recommended by the BioSemi EEG User Guide (±25 μV). A horizontal and vertical electrooculogram (EOG) was recorded bipolarly using electrodes placed below and above the right eye and at the outer canthus of each eye.

EEG data were analyzed using EMEGS software (Peyk et al., 2011). The EEG signal was offline filtered using 0.1 Hz high‐pass and 40 Hz low‐pass zero‐phase filters and then re‐referenced to linked‐mastoid channels. Ocular artifacts were corrected using the Biosig toolbox; artifact rejection was then conducted using statistical control of artifacts in high‐density EEG/MEG data (Junghöfer et al., 2000). This procedure (1) detects individual channel artifacts, (2) detects global artifacts, (3) replaces artifact‐contaminated channels with spline interpolation, and (4) computes the variance of the signal across trials to document the stability of the averaged waveform. The artifact rejection relied on calculating statistical parameters for the absolute measured amplitudes of potentials over time, their standard deviation, and the determination of boundaries for each parameter. If noisy channels were discovered, their signal was estimated by spherical spline interpolation based on the weighted signal of all remaining sensors. A minimum threshold of 0.2 for the estimated goodness of interpolation was employed, and trials which exceeded this value were rejected. If more than 30% of the trials in any of the four EEG recordings (PreEx, PostEx, PreRest, PostRest) were rejected, the respective participant would have been excluded from further analysis. However, no participant had to be excluded due to global cross‐conditional EEG artifacts. Epochs of 200 ms before stimulus onset and 1000 ms post‐stimulus onset were then extracted from the continuous EEG and baseline corrected according to the 150 ms time window before stimulus onset. Stimulus‐locked ERP averages were computed separately for each participant for the positive and negative pictures and experimental conditions. A 2 × 2 × 2 × 2 ANOVA with a 2‐level within‐subject factor PROTOCOL (EX, REST), a 2‐level within‐subject factor TIME (PRETEST, POSTEST), a 2‐level within‐subject factor VALENCE (POS, NEG), and a 2‐level between‐subject factor GROUP (DEP, HC) revealed a significant effect of TIME [F(1,46) = 8.35, *p* = .006, *η* ^2^ = 0.15]. The number of remaining (artifact‐free) trials was lower for POST‐TEST than for PRE‐TEST. The number of remaining trials did not differ between groups [*F*(1,46) = 0.15, *p* = .76, *η* ^2^ = 0.02], between positive and negative pictures [*F*(1,46) = 2.16, *p* = .15, *η* ^2^ = 0.05], or between the Rest and Exercise protocols [*F*(1,46) = 1.40, *p* = .24, *η* ^2^ = 0.03]. Importantly, the analysis also did not reveal any significant interaction of the tested factors [all *F* < 1.42, all *p* > .24, all *η* ^2^ < 0.03]. The mean number of remaining trails by conditions (max. 240) after EEG artifact rejection is presented in Table 2.

**TABLE 2:** The mean number of trials included in the analysis by experimental conditions (max. 240)

	Pre‐test (M ± SD)	Post‐test (M ± SD)
Positive pictures		
EX DEP	221.87 ± 11.92	217.17 ± 10.59
EX HC	221.13 ± 1.18	218.71 ± 10.71
REST DEP	220.50 ± 8.04	217.91 ± 9.70
REST HC	219.33 ± 9.36	216.42 ± 9.94
Negative pictures		
EX DEP	218.80 ± 10.73	215.25 ± 11.33
EX HC	220.71 ± 9.37	219.29 ± 9.06
REST DEP	217.79 ± 11.89	216.04 ± 10.57
REST HC	219.42 ± 9.80	214.46 ± 8.45

Abbreviations: EX DEP, exercise protocol, depression group; EX HC, exercise protocol, healthy control group; REST DEP, rest protocol, depression group; REST HC, rest protocol, healthy control group.

### 2.7 LPP

The late positive potential (LPP) was used as a measure of emotional reactivity to the presented pictures. Based on previous studies on the effects of exercise on emotional reactivity in depressed participants (Brush et al., 2020, 2021), we a priori defined LPP as the mean voltage at the Pz electrode within the 400–1000 ms time window following picture presentation. Relevant values for positive and negative pictures for each condition and group were exported for further statistical analysis.

### 2.8 Data analysis

All statistical analyses were performed using SPSS Statistics version 21 (IBM, Armonk, NY, USA) and JASP version 0.14.1 (JASP team 2020) software.

#### 2.8.1 Exercise intervention manipulation

To test the effectiveness of the experimental manipulation, we used 2 × 2 repeated analyses of variance (ANOVAs). Each ANOVA was calculated separately for the mean heart rate (HR) and the rate of perceived exertion (RPE) measured during both experimental protocols. Each ANOVA consisted of a 2‐level within‐subject factor PROTOCOL (levels: EX, REST) and a 2‐level between‐subject factor GROUP (levels: DEP, HC). We were particularly interested in the PROTOCOL effect (which reflects the effect of the experimental manipulation) and the PROTOCOL by GROUP interaction (to learn if the experimental manipulation had a similar impact on HR and RPE across the two tested groups). All of the pairwise comparisons were corrected using the Bonferroni method.

#### 2.8.2 Self‐reported mood

To test the influence of the exercise on self‐reported mood, 2 × 2 × 2 ANOVAs were performed separately for each scale of the POMS (TEN, ANG, FAT, DEP, CON, ERA, VIG, TMD) and SEES (PWD, PD, F) questionnaires. Each ANOVA consisted of a 2‐level within‐subject factor PROTOCOL (EX, REST), a 2‐level within‐subject factor TIME (PRETEST, POSTEST), and a 2‐level between‐subject factor GROUP (DEP, HC). We were particularly interested in the effects of GROUP (which reflects potential differences in experienced mood across DEP and HC groups); PROTOCOL*TIME (which reflects the effect of the exercise protocol on mood), and GROUP*PROTOCOL*TIME (which reflects the potentially different effects of exercise on mood across DEP and HC groups). Additionally, pairwise planned comparisons were utilized to specifically test the hypothesis regarding the change of mood after vs. before the experimental protocol. All the pairwise comparisons were corrected using the Bonferroni method.

#### 2.8.3 LPP data

To test the influence of the exercise on LPP amplitude, a 2 × 2 × 2 × 2 ANOVA was performed with a 2‐level within‐subject factor PROTOCOL (EX, REST), a 2‐level within‐subject factor TIME (PRETEST, POSTEST), a 2‐level within‐subject factor VALENCE (POS, NEG), and a 2‐level between‐subject factor GROUP (DEP, HC). We were particularly interested in the following effects: VALENCE (to confirm that positive and negative pictures did not differ in terms of induced emotional reactivity); GROUP (to test for distinct emotional reactivity between DEP and HC groups); VALENCE*GROUP (to test if emotional reactivity to positive or negative pictures differed between DEP and HC groups); PROTOCOL*TIME (to test the overall effects of exercise on emotional reactivity); PROTOCOL*TIME*GROUP (to test if the effects of exercise differed across the two tested groups); PROTOCOL*TIME*VALENCE*GROUP (to test if the effects of exercise differently affected positive and negative picture processing between the DEP and HC groups). Additionally, pairwise planned comparisons were utilized to specifically test the hypothesis regarding the change of LPP in response to positive and negative pictures after vs. before exercise. All the pairwise comparisons were corrected using the Bonferroni method.

Moreover, to simplify the visualization of the results and enable further exploratory analyses, we reduced the TIME factor by computing the LPP subtraction‐based difference scores. Specifically, the difference scores were computed as the *LPP post‐experimental protocol* minus the *LPP pre‐experimental protocol* (POSTTEST – PRETEST), separately for each group (DEP, HC), experimental protocol (EX, REST), and valence of pictures (POS, NEG).

The measures calculated above are referred to as LPP_diff for the rest of the manuscript. LPP_diff represents the effect of the experimental session on LPP such that negative values of LPP_diff indicate a decrease of the magnitude of LPP due to the experimental protocol. In contrast, positive values indicate an increase in the magnitude of LPP due to the experimental protocol. Similarly, mood reactivity to exercise was calculated as difference scores for each scale of the POMS and SESS questionnaires (POMS_diff and SEES_diff, respectively). The difference scores for each scale were calculated by subtracting each scale's score after the experimental protocols from the score before the experimental protocols.

Finally, we performed an additional exploratory analysis to explore if changes in emotional responsiveness after exercise depend on additional measures acquired in this study. LPP_diff in response to positive and negative pictures was correlated with participants' characteristics (age, BMI, VO_2_max, HRSD, SDPARQ, sRAMP, STAI, ERQ, and PSS); the measures of mood reactivity collected during the study (POMS_diff and SEES_diff scales); and ratings of the pictures used in the study (valence and arousal for positive and negative pictures). Due to the explanatory nature of these analyses and the multiple testing that was performed, the relevant *p* values of all correlation tests were subjected to the false discovery rate correction (FDR) procedure (FDR = 0.05; total number of tests = 36) (Benjamini & Hochberg, 1995).

## 3 RESULTS

### 3.1 Exercise intervention manipulation

The PROTOCOL showed a significant effect on mean heart rates (HR) [*F*(1,46) = 2226.75, *p* < .001, *η* ^2^ = 0.98] and rates of perceived exertion (RPE) [*F*(1,46) = 942.12, *p* < .001, *η* ^2^ = 0.95]. The RPE and HR measures were lower for the REST condition as compared with the EX condition (*p* < .001).

The analysis showed no significant PROTOCOL*GROUP interaction effect on mean HR [*F*(1,46) = 0.02, *p* = .88] or RPE [*F*(1,46) = 0.01, *p* = .91]. The RPE and HR measures for the REST and EX conditions were not significantly different across the two tested groups (*p* = .57 and *p* = .88 respectively). The characteristics of the workloads during the experimental protocols are presented in Table 3.

**TABLE 3:** Characteristics of experimental sessions (M ± SD) by groups

	EX	REST
RPE (Borg Scale)		
Depression	12.33 ± 1.20	6.33 ± 0.48
Healthy control	12.25 ± 1.26	6.20 ± 0.42
Total	12.29 ± 1.22	6.27 ± 0.45
RPE (verbal anchor)		
Depression	“Light/Somewhat hard”	“No exertion at all”
Healthy control	“Light/Somewhat hard”	“No exertion at all”
Total	“Light/Somewhat hard”	“No exertion at all”
Mean HR (bpm)		
Depression	143.24 ± 8.91	70.62 ± 10.18
Healthy control	142.69 ± 7.92	71.88 ± 10.69
Total	142.98 ± 8.34	71.25 ± 10.35
%HRmax		
Depression	75.08 ± 4.79	36.97 ± 4.95
Healthy control	74.78 ± 4.42	37.65 ± 5.37
Total	74.94 ± 4.56	37.31 ± 5.18
%HRR		
Depression	60.41 ± 6.89	n/a
Healthy control	59.44 ± 6.46	n/a
Total	59.92 ± 6.62	n/a
Mean power (Watt)		
Depression	80.66 ± 22.52	n/a
Healthy control	87.29 ± 29.52	n/a
Total	83.96 ± 26.19	n/a

Abbreviations: EX, moderate‐intensity exerciseHR, heart rate; HRR, heart rate reserve; HRmax, maximum heart rate; REST, seated rest condition; RPE, rate of perceived exertion.

### 3.2 Self‐reported mood

The mean responses for each of the questionnaire scales that were assessed before (pre‐test) and after (post‐test) the experimental protocols are shown separately for each PROTOCOL (EX, REST) and each group (DEP, HC) in Table 4.

**TABLE 4:** POMS and SEES questionnaires results

	Pre‐test (M ± SD)	Post‐test (M ± SD)	Difference (post – Pre) (M ± SD)
Total mood disturbances (TMD, POMS)^a^ ^,^ ^b^			
EX DEP	128.83 ± 21.23	119.04 ± 17.06	−9.79 ± 16.83*
EX HC	99.63 ± 9.91	95.46 ± 8.51	−4.17 ± 7.74*
REST DEP	132.25 ± 24.88	132.75 ± 21.63	0.50 ± 9.46
REST HC	99.79 ± 8.84	102.25 ± 11.43	2.46 ± 7.38
Tension (TEN, POMS)^a^			
EX DEP	11.08 ± 5.36	9.71 ± 4.61	−1.38 ± 2.78*
EX HC	6.92 ± 1.25	6.54 ± 0.88	−0.38 ± 1.17
REST DEP	12.00 ± 5.52	11.08 ± 5.65	−0.92 ± 2.66*
REST HC	7.04 ± 1.49	6.63 ± 1.10	−0.42 ± 0.78
Anger (ANG, POMS)^a^			
EX DEP	7.63 ± 2.90	7.00 ± 2.06	−0.63 ± 1.58*
EX HC	6.41 ± 1.02	6.25 ± 0.90	−0.17 ± 0.38
REST DEP	9.17 ± 5.22	8.75 ± 4.41	−0.42 ± 1.95
REST HC	6.54 ± 1.25	6.25 ± 0.74	−0.29 ± 0.81
Fatigue (FAT, POMS)^a^			
EX DEP	10.50 ± 5.54	10.96 ± 3.67	0.46 ± 4.92
EX HC	6.46 ± 1.67	7.25 ± 2.35	0.79 ± 2.38
REST DEP	9.58 ± 4.18	11.13 ± 4.19	1.54 ± 3.51*
REST HC	6.17 ± 1.27	6.63 ± 1.86	0.46 ± 1.82
Depression (DEP, POMS)^a^ ^,^ ^b^			
EX DEP	12.83 ± 6.25	10.36 ± 5.08	−2.46 ± 4.26*
EX HC	7.68 ± 1.05	7.21 ± 0.72	−0.46 ± 0.78
REST DEP	14.04 ± 6.91	13.83 ± 6.40	−0.21 ± 2.48
REST HC	7.50 ± 0.98	7.88 ± 1.80	0.38 ± 1.31
Esteem‐related affect (ERA, POMS) ^a,b^			
EX DEP	13.13 ± 3.28	15.04 ± 3.95	1.92 ± 2.57*
EX HC	17.86 ± 3.39	19.63 ± 3.28	1.75 ± 3.04*
REST DEP	13.08 ± 3.39	12.71 ± 2.88	−0.38 ± 2.56
REST HC	17.92 ± 3.20	17.29 ± 3.68	−0.63 ± 2.32
Vigor (VIG, POMS)^a^ ^,^ ^b^			
EX DEP	10.08 ± 3.84	13.00 ± 4.45	2.92 ± 3.48*
EX HC	17.04 ± 4.37	18.13 ± 4.00	1.08 ± 2.90*
REST DEP	10.08 ± 4.11	8.96 ± 3.77	−1.13 ± 2.89*
REST HC	16.08 ± 4.11	13.67 ± 4.47	−1.42 ± 2.62*
Confusion (CON, POMS)^a^			
EX DEP	10.00 ± 3.04	9.04 ± 2.97	−0.96 ± 2.27*
EX HC	7.08 ± 2.26	5.96 ± 1.57	−1.13 ± 1.73*
REST DEP	10.63 ± 3.49	9.63 ± 3.44	−1.00 ± 1.75*
REST HC	6.54 ± 1.62	6.83 ± 2.16	0.29 ± 1.65
Positive well‐being (PWB, SEES)^a^ ^,^ ^b^			
EX DEP	11.04 ± 5.56	13.17 ± 6.04	2.13 ± 3.44*
EX HC	19.00 ± 4.33	20.54 ± 4.09	1.54 ± 2.87*
REST DEP	10.58 ± 4.57	9.33 ± 4.54	−1.25 ± 2.01*
REST HC	18.71 ± 3.50	18.33 ± 3.52	−0.36 ± 2.92
Psychological distress (PD, SEES)^a^ ^,^ ^b^			
EX DEP	9.33 ± 6.53	6.71 ± 4.10	−2.63 ± 4.33*
EX HC	4.88 ± 2.64	4.29 ± 0.86	−0.58 ± 1.95
REST DEP	9.63 ± 6.12	8.54 ± 5.13	−1.08 ± 2.43
REST HC	4.58 ± 1.64	4.50 ± 1.22	−0.08 ± 1.38
Fatigue (F, SEES)^a^			
EX DEP	11.86 ± 7.34	11.96 ± 5.11	0.08 ± 5.42
EX HC	6.83 ± 2.92	7.54 ± 3.36	0.71 ± 4.01
REST DEP	11.17 ± 6.04	12.54 ± 5.66	1.38 ± 4.02
REST HC	6.25 ± 2.75	6.96 ± 3.94	0.71 ± 2.77

Abbreviations: DEP, depression group; EX, moderate‐intensity exercise; HC, healthy control group; REST, seated rest condition.

^a^ Significant GROUP effect (DEP vs HC).

^b^ Significant PROTOCOL (EX vs REST) × TIME (PRETEST vs POSTEST) interaction.

* Significant difference as measured by planned comparison post vs pretest at level *p* < .05.

#### 3.2.1 Total mood disturbances (TMD, POMS)

The analyses revealed a significant effect of GROUP [*F*(1,46) = 45.90, *p* < .001, *η* ^2^ = 0.50]. The average score on the TMD scale was significantly higher [*t =* 4.27*; p* < .001] for the DEP group [*M* = 128.22; *SD* = 21.76] than for the HC group [*M* = 99.28; *SD* = 9.90] group. There was a significant PROTOCOL*TIME interaction [*F*(1,46) = 15.00, *p* < .001, *η* ^2^ = 0.25]. As compared with the pre‐test, the average score on the TMD scale decreased significantly [*t = −*6.98*; p* < .001] after the EX protocol [*M* = 107.25; *SD* = 17.88; *M* = 114.23; *SD* = 22.06; for post‐test and pre‐test, respectively], whereas it did not significantly change after the REST protocol [*t =* 0.93*; p* = .36] [*M* = 117.50; *SD* = 23.03; *M* = 116.02; *SD* = 24.79; for post‐test and pre‐test, respectively]. The GROUP*PROTOCOL*TIME interaction was non‐significant.

#### 3.2.2 Tension (TEN, POMS)

The analyses revealed a significant effect of GROUP, [*F*(1,46) = 16.89, *p* < .001, *η* ^2^ = 0.27]. The average score on the TEN scale was significantly higher [*t =* 4.11*; p* < .001] for the DEP group [*M* = 10.97; *SD* = 5.28] than for the HC group [*M* = 6.78; *SD* = 1.20]. The PROTOCOL*TIME and GROUP*PROTOCOL*TIME interactions were non‐significant.

#### 3.2.3 Anger (ANG, POMS)

The analyses revealed a significant effect of GROUP [*F*(1,46) = 6.62, *p* = .013, *η* ^2^ = 0.13]. The average score on the ANG scale was significantly higher [*t =* 2.57*; p* = .013] for the DEP group [*M* = 8.14; *SD* = 3.89] than for the HC group [*M* = 6.36; *SD* = 0.96]. The PROTOCOL*TIME and GROUP*PROTOCOL*TIME interactions were non‐significant.

#### 3.2.4 Fatigue (FAT, POMS)

The analyses revealed a significant effect of GROUP, [*F*(1,46) = 24.49, *p* < .001, *η* ^2^ = 0.35]. The average score on the FAT scale was significantly higher [*t =* 4.95*; p* < .001] for the DEP group [*M* = 10.54; *SD* = 4.42] than for the HC group [*M* = 6.63; *SD* = 1.84] group. The PROTOCOL*TIME and GROUP*PROTOCOL*TIME interactions were non‐significant.

#### 3.2.5 Depression (DEP, POMS)

The analyses revealed a significant effect of GROUP [*F*(1,46) = 19.42, *p* < .001, *η* ^2^ = 0.30]. The average score on the DEP scale was significantly higher [*t =* 4.41*; p* < .001] for the DEP group [*M* = 12.77; *SD* = 6.27] than for the HC group [*M* = 7.56; *SD* = 1.21]. There was a significant PROTOCOL*TIME interaction [*F*(1,46) = 8.47, *p* = .006, *η* ^2^ = 0.16]. As compared with the pre‐test, the average score on the DEP scale decreased significantly [*t = −*3.91*; p* < .001] after the EX protocol [*M* = 8.79; *SD* = 3.93; *M* = 10.25; *SD* = 5.15; for post‐test and pre‐test, respectively], whereas it did not significantly change after the REST protocol [*t =* 0.22*; p* = .82] [*M* = 10.85; *SD* = 5.54; *M* = 10.77; *SD* = 5.90 for post‐test and pre‐test, respectively]. The GROUP*PROTOCOL*TIME interaction was non‐significant.

#### 3.2.6 Esteem‐Related affect (ERA, POMS)

The analyses revealed a significant effect of GROUP [*F*(1,46) = 31.89, *p* < .001, *η* ^2^ = 0.41]. The average score on the ERA scale was significantly lower [*t = −*5.65*; p* < .001] for the DEP group [*M* = 13.49; *SD* = 3.46] than for the HC group [*M* = 18.18; *SD* = 3.45] group. There was a significant PROTOCOL*TIME interaction [*F*(1,46) = 20.15, *p* < .001, *η* ^2^ = 0.31]. As compared with the pre‐test, the average score on the ERA scale decreased significantly [*t = −*4.81*; p* < .001] after the EX protocol [*M* = 17.33; *SD* = 4.27; *M* = 15.50; *SD* = 4.08 for post‐test and pre‐test, respectively], whereas it did not significantly change after the REST protocol [*t =* −1.31*; p* = .19] [*M* = 15.00; *SD* = 4.01; *M* = 15.50; *SD* = 4.07; for post‐test and pre‐test, respectively]. The GROUP*PROTOCOL*TIME interaction was non‐significant.

#### 3.2.7 Vigor (VIG, POMS)

The analyses revealed a significant effect of GROUP [*F*(1,46) = 33.23, *p* < .001, *η* ^2^ = 0.42]. The average score on the VIG scale was significantly [*t = −*5.75*; p* < .001] lower for the DEP group [*M* = 10.53; *SD* = 4.16] than for the HC group [*M* = 16.48; *SD* = 4.36] group. There was a significant PROTOCOL*TIME interaction [*F*(1,46) = 27.31, *p* < .001, *η* ^2^ = 0.37]. As compared with the pre‐test, the average score on the VIG scale increased significantly [*t =* 2.00*; p* < .001] after the EX protocol [*M* = 15.56; *SD* = 4.93; *M* = 13.56; *SD* = 5.38; for post‐test and pre‐test, respectively], whereas it decreased significantly after the REST protocol [*t =* −1.27*; p* = .006] [*M* = 11.81; *SD* = 5.01; *M* = 13.08; *SD* = 4.90; for post‐test and pre‐test, respectively]. The GROUP*PROTOCOL*TIME interaction was non‐significant.

#### 3.2.8 Confusion (CON, POMS)

The analyses revealed a significant effect of GROUP, [*F*(1,46) = 22.16, *p* < .001, *η* ^2^ = 0.33]. The average score on the CON scale was significantly higher [*t =* 4.71*; p* < .001] for the DEP group [*M* = 9.82; *SD* = 3.24] than for the HC group [*M* = 6.60; *SD* = 1.94] group. The PROTOCOL*TIME and GROUP*PROTOCOL*TIME interactions were non‐significant.

#### 3.2.9 Positive Well‐Being (PWB, SEES)

The analyses revealed a significant effect of GROUP [*F*(1,46) = 48.12, *p* < .001, *η* ^2^ = 0.51]. The average score on the PWB scale was significantly lower [*t = −*8.16*; p* < .001] for the DEP group [*M* = 11.03; *SD* = 5.31] than for the HC group [*M* = 19.15; *SD* = 3.91] group. There was a significant PROTOCOL*TIME interaction [*F*(1,46) = 21.25, *p* < .001, *η* ^2^ = 0.32]. As compared with the pre‐test, the average score on the PWB scale increased significantly [*t =* 4.48*; p* < .001] after the EX protocol [*M* = 16.85; *SD* = 6.32; *M* = 15.02; *SD* = 6.37; for post‐test and pre‐test, respectively], whereas it decreased significantly after the REST protocol [*t =* −1.97*; p* = .052] [*M* = 13.833; *SD* = 6.07; *M* = 14.65; *SD* = 5.75; for post‐test and pre‐test, respectively]. The GROUP*PROTOCOL*TIME interaction was non‐significant.

#### 3.2.10 Psychological distress (PD, SEES)

The analyses revealed a significant effect of GROUP [*F*(1,46) = 13.80, *p* < .001, *η* ^2^ = 0.23]. The average score on the PD scale was significantly higher [*t =* 3.72*; p* < .001] for the DEP group [*M* = 8.55; *SD* = 5.58] than for the HC group [*M* = 4.56; *SD* = 1.71] group. There was a significant PROTOCOL*TIME interaction [*F*(1,46) = 12.51, *p* < .014, *η* ^2^ = 0.12]. As compared with the pre‐test, the average score on the PD scale decreased significantly [*t = −*4.03*; p* < .001] after the EX protocol [*M* = 5.50; *SD* = 3.18; *M* = 7.10; *SD* = 5.42; for post‐test and pre‐test, respectively], whereas it did not significantly change after the REST protocol [*t =* −1.47*; p* = .15] [*M* = 6.52; *SD* = 4.22; *M* = 7.10; *SD* = 5.11; for post‐test and pre‐test, respectively]. The GROUP*PROTOCOL*TIME interaction was non‐significant.

#### 3.2.11 Fatigue (F, SEES)

The analyses revealed a significant effect of GROUP, [*F*(1,46) = 19.92, *p* < .001, *η* ^2^ = 0.29]. The average score on the F scale was significantly higher [*t =* 4.35*; p* < .001] for the DEP group [*M* = 11.89; *SD* = 6.02] than for the HC group [*M* = 6.90; *SD* = 3.26]. The PROTOCOL*TIME and GROUP*PROTOCOL*TIME interactions were non‐significant.

### 3.3 LPP data

**FIGURE 2:**
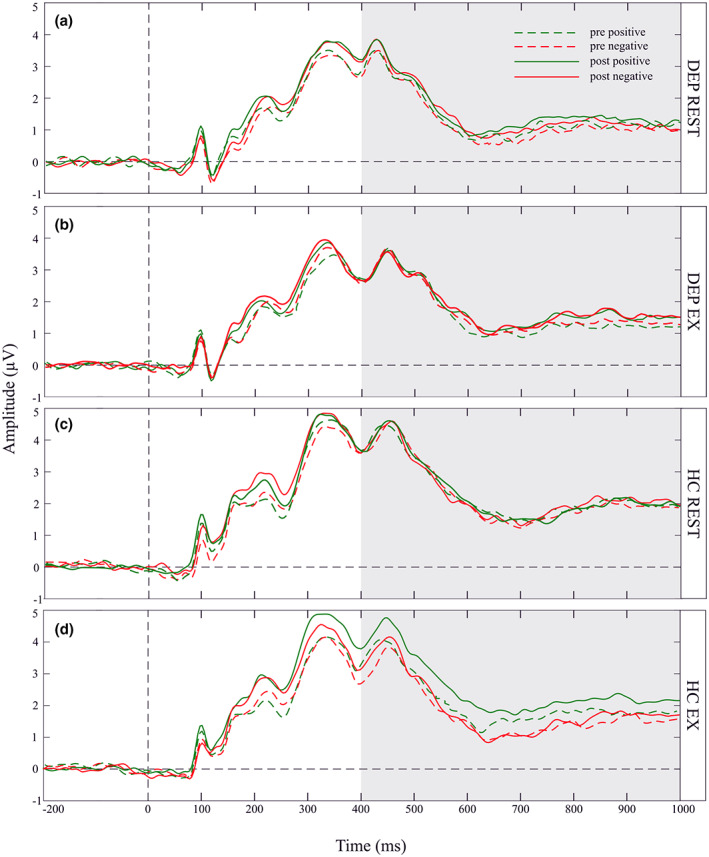
Grand‐average ERP waveforms. Grand‐averaged ERP waveforms at electrode Pz are separately depicted in the four panels: (a) depression group during the rest protocol (DEP REST); (b) depression group during the exercise protocol (DEP EX); (c) healthy control group during the rest protocol (HC REST); and (d) healthy control group during the exercise protocol (HC EX). Each panel depicts waveforms in response to positive (green line) and negative (red line) pictures, which were assessed either before a protocol (dotted line), or after a protocol (solid line). The LPP was scored as the mean amplitude in the time window from 400 to 1000 ms following picture onset (shaded area).

The mean amplitudes of LPP in response to positive and negative pictures measured before (pre‐test) and after (post‐test) the experimental protocols are shown separately for each PROTOCOL (EX, REST) and each group (DEP, HC) in Table 5. Figure 2 shows the corresponding grand‐averaged waveforms for all conditions for the DEP and HC groups.

**TABLE 5:** Mean LPP data (voltage)

	Pre‐test (M ± SD)	Post‐test (M ± SD)	Difference (post – Pre) (M ± SD)
Positive pictures			
EX DEP	1.23 ± 1.30	1.29 ± 1.31	0.06 ± 0.66
EX HC	1.78 ± 1.18	2.27 ± 1.37	0.49 ± 1.01*
REST DEP	1.31 ± 1.19	1.36 ± 1.21	0.05 ± 0.47
REST HC	1.95 ± 1.18	1.97 ± 1.01	0.02 ± 0.63
Negative pictures			
EX DEP	1.22 ± 1.34	1.34 ± 1.26	0.12 ± 0.55
EX HC	1.79 ± 1.16	1.93 ± 1.31	0.14 ± 1.01
REST DEP	1.20 ± 1.17	1.27 ± 1.18	0.07 ± 0.47
REST HC	1.93 ± 1.02	1.99 ± 1.02	0.06 ± 0.52

Abbreviations: DEP, depression group; EX, moderate‐intensity exercise; HC, healthy control group; REST, seated rest condition.

* Significant difference as measured by planned comparison post vs pretest at level *p* < .05.

Analysis revealed a significant effect of GROUP [*F*(1,46) = 4.42, *p* = .04, *η* ^2^ = 0.09]. The average amplitude of LPP in response to pictures was significantly lower [*t =* −2.10*; p* = .04] for the DEP group [*M* = 1.28; *SD* = 1.19] than for the HC group [*M* = 1.95; *SD* = 1.03]. The VALENCE*GROUP, PROTOCOL*TIME, and PROTOCOL*TIME*GROUP interactions were non‐significant.

**FIGURE 3:**
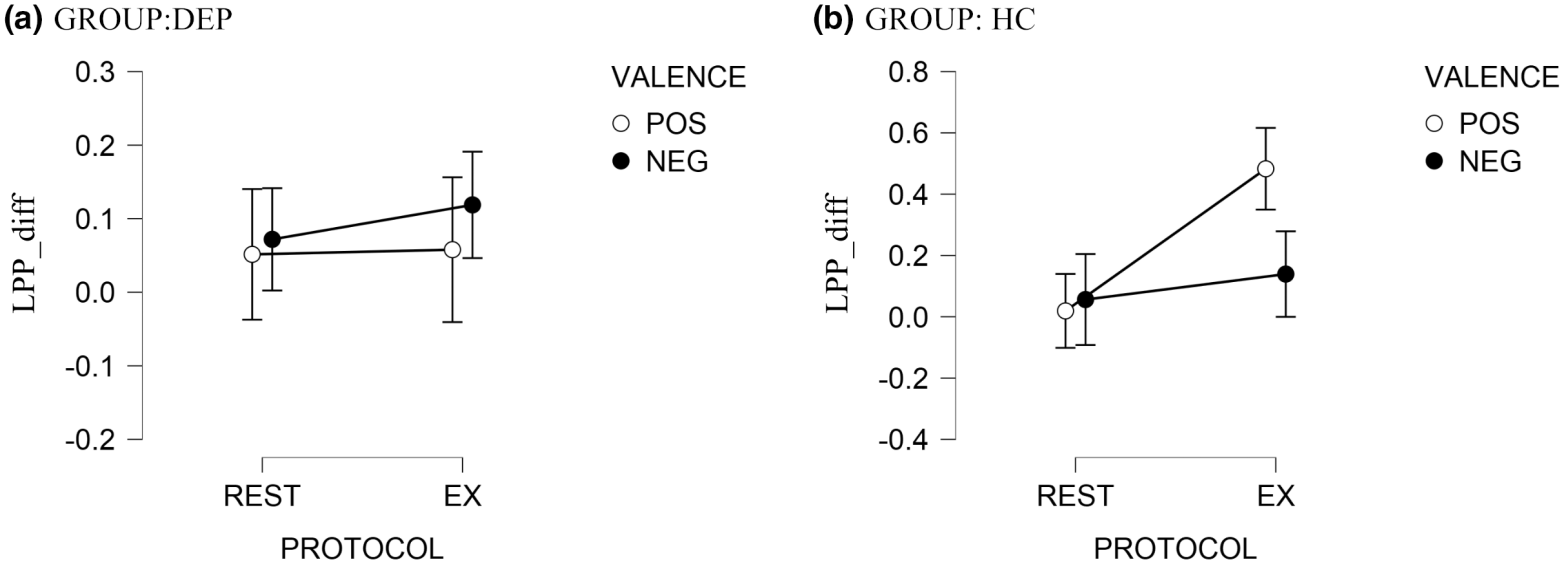
The effects of moderate‐intensity exercise (EX) versus seated REST (REST) on changes in LPP in response to positive and negative pictures. Y‐axis represents LPP_diff, calculated as the difference score of the *LPP post‐experimental protocol* minus the *LPP pre‐experimental protocol*. LPPs increased more for positive pictures in the HC compared to the DEP group, while changes in LPP for negative pictures did not differ across groups. Error bars represent standard errors. DEP, depression group; HC, healthy control group. POS, positive pictures; NEG, negative pictures.

There was a significant PROTOCOL*TIME*VALENCE*GROUP interaction [*F*(1,46) = 4.57, *p* = .04, *η* ^2^ = 0.09]. As compared with the pre‐test, the average amplitude of LPP increased significantly [*t =* 3.33*; p* < .001] after the EX protocol, but only for the HC group, and only in response to pictures of positive VALENCE [*M* = 2.27; *SD* = 1.37; *M* = 1.78; *SD* = 1.18; for post‐test and pre‐test, respectively]. The post‐test vs pre‐test differences in the mean amplitude of LPP were not significant for any other combinations of factor levels [*p* > .05]. The difference scores of the LPP (LPP during posttest minus LPP during pretest, LPP_diff) for each GROUP, PROTOCOL, and VALENCE are presented in Table 5 and Figure 3.

We ran further correlation analyses to determine whether LPP_diff in response to positive and negative pictures for the EX protocol is associated with the participants' characteristics, mood reactivity to exercise (POMS_diff, SEES_diff), or participants' ratings of pictures.

The correlation of LPP_diff in response to positive pictures with the remaining variables was significant only for the score on the Hamilton Rating Scale for Depression (HRSD) [*rho* = −0.36; *p* = .003]. None of the remaining correlations were statistically significant. The correlations between LPP_diff in response to negative pictures and the remaining variables were not significant.

## 4 DISCUSSION

This study investigated the effects of acute aerobic exercise on mood and emotional reactivity in depressed (DEP) and healthy young adults (HC). Self‐reported mood and neural reactivity to emotional pictures (as indexed by the late positive potential, LPP) were assessed before and after two experimental protocols: (1) exercise protocol (EX, 36 min of moderate‐intensity exercise); (2) seated rest condition (REST, 36 min). In line with our predictions, exercise improved self‐reported mood in both groups. The exercise was also related to positive changes in neural emotional reactivity. However (contrary to our expectations), this effect was limited to the HC group. Additional analyses revealed that exercise‐related changes in emotional reactivity are associated with the severity of depressive symptoms across both groups: the effectiveness of exercise in benefiting emotional reactivity decreases as the severity of depressive symptoms increases. Below, we discuss the obtained findings.

### 4.1 Self‐reported mood

The effects of exercise on mood were assessed using POMS and SEES questionnaires, which were administered before and after the two experimental protocols. Analyses included testing for differences in mood between the two groups and testing for the effects of the experimental protocols on mood. In the first case, the DEP group reported generally lowered mood, regardless of the measurement time and experimental protocols. Significant differences concerned all of the eight POMS subscales and all of the three SEES subscales: the DEP group scored higher on negatively valenced scales (Total Mood Disturbances (TMD), Tension (TEN), Anger (ANG), Fatigue (FAT), Depression (DEP), Confusion (CON), and Psychological Distress (PD)); the DEP group scored lower on positively valenced scales (Vigor (VIG), Esteem‐related Affect (ERA), and Psychological Well‐Being (PWB)). In this regard, the results confirm the presence of mood disturbances in the DEP group.

Regarding the impact of the experimental protocols on mood, exercise positively influenced most of the POMS and SEES scales across both tested groups, which was in line with the expectations. Specifically, the exercise protocol decreased scores on the Total mood disturbances (TMD), Depression (DEP), and Psychological Distress (PD) scales; it increased scores on the Vigor (VIG), Esteem‐Related Affect (ERA), and Positive Well‐Being (PWB) scales. At the same, the seated rest condition (REST) either did not influence mood (DEP, ERA, TMD, PD scales) or negatively influenced it (relative decreases in the VIG and PWD scales). The positive effects on mood of the experimental protocols were not different across the two tested groups. However, the pairwise comparisons that were performed regardless of the insignificant effect of the PROTOCOL*TIME interaction showed some differences in how exercise‐influenced mood across the two groups. The exercise protocol decreased scores on the Depression (DEP) and Total Mood Disturbances (TMD) scales only in the DEP group. In contrast, exercise did not change the DEP and TMD scales scores in the HC group. This effect might suggest that exercise had a more profound effect on mood for the DEP group. It has to be remembered, though, that the HC group scored relatively low on the DEP and TMD scales, so an exercise‐induced decrease in negative feelings would barely have been possible for this group (due to the floor effect) (Ligeza et al., 2021). Therefore, in the DEP group, exercise additionally influenced depressive mood, indicating the legitimacy of using exercise to improve mood in people with symptoms of depression.

Overall, the mood pattern results further support previous ample evidence on the beneficial effect of exercise on mood in healthy young adults (for a review and meta‐analysis, see: Asmundson et al. (2013); Reed and Ones (2006)). As for depressed individuals, our results support previous findings indicating the beneficial effect of exercise for both positive and negative mood states, as observed by Brupbacher et al. (2021) and Niedermeier et al. (2021). Our results at least partly support studies showing beneficial effects of exercise that are limited to positive (Bartholomew et al., 2005; Frühauf et al., 2016; Legrand et al., 2018) or negative (Meyer et al., 2016) mood scales. Finally, our results contradict studies in which the effect of exercise was not greater than the effect of the control condition (Meyer et al., 2019; Perez et al., 2020). When interpreting these inconsistencies, attention should be paid to the nature of the control conditions utilized. In the present study and the studies of Brupbacher et al. (2021) and Niedermeier et al. (2021), the control condition (usually reading) did not change or even negatively influenced self‐reported mood. In contrast, in the studies of Bartholomew et al. (2005), Meyer et al. (2016), Meyer et al. (2019), and Perez et al. (2020), a control condition (usually quiet rest) significantly improved mood. As such, the different control conditions across these studies could have affected the interpretation of the exercise effects. For example, the quiet rest protocol could have had a greater anxiolytic and anti‐depressive effect than the reading control protocol, thus reducing the “net” effect of the exercise. This indicates a significant issue with selecting an optimal control condition to study the effects of exercise. The not entirely consistent findings across different studies may also be a result of the various tools used to measure mood. Considering that we used the same or similar instruments as Bartholomew et al. (2005), Legrand et al. (2018), and Meyer et al. (2016), it seems that the effects observed in the control condition may be a more important factor in the observed inconsistencies. Nevertheless, our results strongly support the cumulative evidence regarding the positive effect of exercise on mood states in depression.

### 4.2 Effects of exercise on emotional neural reactivity

One of our main goals was to assess the effects of exercise on emotional neural responsiveness in healthy and depressed adults. For this purpose, we presented participants with positive and negative pictures; we then measured their brain responses using a well‐established marker of emotional reactivity: late positive potential (LPP). We expected that exercise would positively influence emotional reactivity in both groups by increasing LPP in response to positive pictures and/or decreasing LPP in response to negative pictures.

In line with the expectations, exercise was associated with improved emotional reactivity in the healthy controls (HC). When comparing post‐test with pre‐test measurements, the exercise protocol increased LPP in response to positive pictures but did not change LPP in response to negative pictures. At the same time, the REST protocol did not change LPP in response to either positive or negative pictures. As such, the results for the HC group support increasing evidence on the beneficial effect of exercise on emotional neural reactivity (Brush et al., 2020, 2021; Ligeza et al., 2020; Schmitt et al., 2019; Tartar et al., 2018).

In previous research, this effect manifested as an increase in positive reactivity (Brush et al., 2020, 2021), a decrease in negative reactivity (Schmitt et al., 2019; Tartar et al., 2018), or both (Ligeza et al., 2020). However, more evidence suggests that exercise is predominantly associated with emotional responsiveness by increasing positive reactivity rather than reducing negative reactivity. In the only study that showed both an increased positive reactivity and a decreased negative reactivity, the effects were more significant for the former than for the latter. Moreover, while two previous studies did not reveal the effects of exercise on negative reactivity (Brush et al., 2020; Thom et al., 2019), only one failed to show effects on positive reactivity (Tartar et al., 2018). Considering the results obtained in this study, it is tempting to conclude that exercise is predominantly associated with an increase in positive reactivity. It might be speculated that exercise‐related positive mood changes cause stimuli consistent with this mood to be processed more intensely, or that exercise mainly changes the processing of appetitive stimuli. Literature in other domains shows that that information is often processed in a *mood‐congruent* manner. For example, people in a positive mood are more likely to recall positive memories (e.g., Bower, 1991; Mayer et al., 1995) and report being more satisfied with their lives (Schwarz & Clore, 1983). Such mood‐congruent effects after exercise were observed in a previous study (Brush et al., 2020), but (as shown below) we failed to observe them.

Regarding the relationships between exercise and emotional reactivity in depressed individuals, we expected a similar positive change in emotional reactivity as in the HC group. However, this hypothesis was not supported by the data. Neither experimental protocol modulated LPP amplitude in response to positive or negative pictures in the DEP group. So far, only two studies have tested the relationship between exercise‐induced changes in emotional reactiveness and depression (Brush et al., 2020, 2021). Brush et al. (2020) reported that exercise was associated with increased reactivity to positive stimuli (increased LPP), regardless of depressive symptoms. This suggested that exercise is equally effective across the spectrum of depressive symptoms; however, in the second study carried out with healthy and depressed participants, an increase in positive responsiveness after exercise was observed only in healthy participants (Brush et al., 2021). This led to the conclusion that depressed individuals may not experience the same changes in positive emotional reactivity as healthy controls, at least not after a single session of exercise. In this regard, our study shed more light on the mixed evidence from previous studies, thus supporting the observation concerning the lack of association between acute exercise and emotional reactiveness in depressed individuals.

The question arises as to why exercise was ineffective in changing emotional responsiveness in depressed individuals in Brush et al. (2021) and the present study. In an attempt to answer this question, Brush et al. (2021) performed additional analyses and divided the depressed group into three subgroups of individuals displaying specific characteristics of depression: a group with lifetime histories of depression (n = 10), a group of persons with intact mood reactivity (n = 18), and a group of persons with impaired mood reactivity (n = 15). These analyses revealed that exercise was effective only in participants with intact mood reactivity, i.e., depressed participants who maintained their normal reactions to usually pleasant stimuli (lifetime history and intact mood groups). Other clinical characteristics, including current depressive symptom severity, failed to moderate the exercise‐related change in positive reactivity.

Here, we also performed additional analysis to explore possible moderators of exercise‐related changes in emotional reactivity. These analyses aimed to verify whether the observed change in emotional reactivity after exercise (referred to here as LPP_diff) was associated with the other measured variables, including selected background characteristics of participants (age, BMI, declared exercise activity levels, depression severity symptoms as measured by HRSD scales, anxiety trait, perceived stress, IQ, and tendencies to control negative emotions), and changes in mood as a result of exercise (referred here as POMS_diff, SEES_diff). Among all the variables, only the severity of depressive symptoms showed significant correlations with LPP_diff in response to positive pictures. The more severe the depressive symptoms, the smaller the exercise‐related benefits in emotional reactivity. In this regard, our results are not entirely consistent with the study of Brush et al. (2021), in which the severity of depressive symptoms failed to moderate LPP_diff.

Additionally, Brush et al. (2021) suggested that mood reactivity might be the only characteristic that moderates this relationship. In the present study, we measured such mood reactivity by assessing mood changes in response to exercise (POMS_diff, SEES_diff); however, these measures showed no correlation with LPP_diff. Nonetheless, both Brush et al. (2021) and the current study indicate that the associations between emotional reactivity and exercise – at least for a single session of exercise – are limited for depressed individuals.

It should also be noted that in the present study, the DEP group (as compared with the HC group) was characterized by overall reduced LPP amplitudes in response to both positive and negative pictures. This observation aligns with the *emotion context insensitivity model* (ECI) of depression, which states that depressed individuals display reduced emotional reactivity to both positive and negative stimuli (Rottenberg et al., 2005). This insensitivity was significantly correlated with the severity of depressive symptoms (as measured by the Hamilton Rating Scale for Depression) in our study.^5^ This suggests that higher levels of depression symptoms are associated with less sensitivity to emotional stimuli. It might be possible that, with decreasing emotional sensitivity, the effects of exercise on emotional reactivity become more and more limited. In this sense, both our study and Brush et al.’s (2021) suggest that exercise might only be associated with benefits in emotional reactivity when emotional/mood reactivity is not severely altered. These studies also suggest that LPP might be a proper neural indicator of the efficacy of exercise in affecting emotional responsiveness.

Notably, this study provides evidence on the relationship between exercise and emotional responsiveness in depressed individuals that is more robust than the previously available evidence. To our knowledge, this is the first EEG study that has utilized both pre‐test and post‐test measurements (c.a. Brush et al., 2020) as well as control and experimental conditions (c.a. Brush et al., 2021). Additionally, we measured emotional reactivity for positive and negative pictures (c.a. Brush et al., 2021) and measured changes in mood accompanying these reactions (c.a. Brush et al., 2021). Finally, the two tested groups were well matched with each other in terms of selected background characteristics.

Interestingly, exercise‐related changes in self‐reported mood were not associated with changes in neural emotional reactivity (LPP) in healthy and depressed groups. Since mood and emotional reactivity both reflect affective experience, it was expected that the beneficial effect of exercise on mood would be associated with corresponding changes in emotional reactivity. Such a mood‐congruent effect of emotional reactivity after exercise was previously reported by Brush et al. (2020), but we did not observe such a relationship. This suggests that the influence of exercise on mood and emotional reactivity might be based on partially distinct mechanisms. Compared with emotions, mood is a more subjective and less dynamic state. As such, the effects of exercise on mood might depend to a greater extent on psychological factors (e.g., expectations and feelings of self‐mastery during the exercise). On the other hand, emotional reactivity (especially as measured by LPP) might depend more on physiological processes (e.g., changes in brain activity and endorphin release). Separate mechanisms of the influence of exercise on mood and emotional responsiveness could also explain why exercise benefited mood in healthy and depressed participants, while exercise was associated with emotional reactiveness only in the healthy group. One exercise session may be sufficient to affect self‐reported mood states in depressed individuals, but it does not seem enough to affect the negative physiological patterns of emotional responsiveness.

It is important to emphasize that we tested the acute effects of only a single exercise session. Future research should also investigate the effect of a longer intervention (e.g., 3 months) during which depressed participants are regularly active. Longer‐lasting interventions, which are usually associated with changes in brain plasticity (Zhao et al., 2020), might reverse the negative pattern of emotional reactivity. Additionally, in this study, we only tested the effects of moderate‐intensity exercise. However, it is important to stress that the exercise intensity used in this study (75% of maximal heart rate) is within the upper limit of moderate‐intensity exercise. It might also be considered vigorous exercise by other standards. It would also be important for future research to assess the effects of different exercise parameters (duration, intensity, and form) on the LPP.

It should also be noted that the additional correlation analyses in this work were conducted on data derived from the entire sample (across the two tested groups). This was motivated by concern for the reliability of the correlation coefficient in small participant samples (Schönbrodt & Perugini, 2013). We believe that 24 participants (experimental group) are insufficient to reliably assess individual differences. In this sense, we cannot determine whether the relationship between exercise and emotional responsiveness depends on specific characteristics within the depression group. Future studies should include more participants to increase the reliability of the correlation analyses, thus allowing conclusions about individual differences in response to exercise among depressed individuals.

Moreover, the correlation and ANOVA analyses are partly redundant as the distribution of the severity of depression symptoms was close to bimodal. However, the multivariate correlation analysis made it possible to exclude the influence of other potential confounding variables (e.g., anxiety or fitness levels and tendency to control negative emotions), as depressive symptoms were the only variable that showed statistically significant associations with the LPP effect. Therefore, we believe that the consistency of the conclusions of these two analyses strengthens the main message of our study.

## 5 CONCLUSIONS

This study confirms the well‐grounded positive effects of acute aerobic exercise on mood; more importantly, it sheds more light on the associations between exercise and neural emotional reactivity in healthy and depressed individuals. Regarding healthy controls, the study indicates that exercise might be associated with positive changes in brain responses to positive but not negative stimuli, supporting emotional reactivity. In depressed individuals, the study indicates that the positive effects of exercise may be limited. Although exercise benefits the mood of depressed participants, it is not associated with their neural emotional responsiveness. It also seems that the association between exercise and emotional responsiveness is related to the severity of depressive symptoms across healthy and depressed populations: the more severe depressive symptoms are, the less likely it is that exercise can improve neural emotional reactiveness.

## AUTHOR CONTRIBUTIONS

**Tomasz S. Ligeza:** Conceptualization; data curation; formal analysis; funding acquisition; investigation; methodology; project administration; resources; software; visualization; writing – original draft; writing – review and editing. **Marcin Maciejczyk:** Methodology; resources; writing – review and editing. **Miroslaw Wyczesany:** Supervision; writing – review and editing. **Markus Junghöfer:** Conceptualization; methodology; software; supervision; writing – review and editing.

## Footnotes

^1^
Referring to the BDI‐II manual (Beck et al., 1996), at least 14 points indicate mild depression. However, our criterion was set to 15 points to avoid near‐borderline results. The criterion for participants without depression was set at 10 points to differentiate the two groups sufficiently in terms of the reported depressive symptoms.

^2^
Based on previous evidence, repetition of emotional picture presentation does not lead to habituation of brain responses that correlate with emotional reactivity (Codispoti et al., 2006; Schupp et al., 2006).

^3^
While some of the pictures were selected from the International Affective Picture System (IAPS; Lang, Bradley, & Cuthbert, 2008, e.g., mutilation category), in order to enable us to balance luminance and complexity, our research questions necessitated more scene contents than were available in the IAPS. In particular, based solely on the IAPS database, we could not match positive and negative pictures in terms of induced emotional arousal. The remaining pictures were selected by the Georgia Emotion Neuro‐Imaging Lab of Prof. Dean Sabatinelli (University of Georgia, USA) from non‐copyrighted photographs available on the internet. These pictures are consistent in composition and content with the IAPS pictures, and they will be considered for future versions of the IAPS (a more generic description of how the pictures were gathered and standardized can be found in Sabatinelli and Frank (2019)). Identical (Junghofer et al., 2017; Ligeza et al., 2020; Winker et al., 2020) or similar (Farkas et al., 2020; Frank & Sabatinelli, 2019) picture sets were used in previous studies. The picture sets are available on request from the Georgia Emotion Neuro‐Imaging Lab (email: sabat@uga.edu).

^4^
Cronbach's α was calculated based on the responses given during the first completion of the questionnaires in this study (pre‐test during the first protocol).

^5^
Our post hoc analyses revealed a significant correlation between mean LPP amplitude and severity of depressive disorder, as measured by the HRSD scale (rho – 0.421 *p* = .003).
